# A comparison of a new multinomial stopping rule with stopping rules of fleming and gehan in single arm phase II cancer clinical trials

**DOI:** 10.1186/1471-2288-11-95

**Published:** 2011-06-21

**Authors:** John R Goffin, Greg R Pond, Dongsheng Tu

**Affiliations:** 1 McMaster University, Juravinski Cancer Centre, 699 Concession St., Hamilton, Ontario, L8V 5C2, Canada; 2 McMaster University, Ontario Clinical Oncology Group (OCOG), Juravinski Hospital G(60) Wing. 1st Floor, 711 Concession Street, Hamilton, Ontario, L8V 1C3, Canada; 3 Dongsheng Tu, NCIC Clinical Trials Group, Queen's University, 10 Stuart Street, Kingston, Ontario, K7L 3N6, Canada

## Abstract

**Background:**

Response rate (RR) alone may be insensitive to drug activity in phase II trials. Early progressive disease (EPD) could improve sensitivity as well as increase stage I stopping rates. This study compares the previously developed dual endpoint stopping rule (DESR), which incorporates both RR and EPD into a two-stage, phase II trial, with rules using only RR.

**Methods:**

Stopping rules according to the DESR were compared with studies conducted under the Fleming (16 trials) or Gehan (23 trials) designs. The RR hypothesis for the DESR was consistent with the comparison studies (*r*_alt _= 0.2, *r*_nul _= 0.05). Two parameter sets were used for EPD rates of interest and disinterest respectively (*epd*_alt_, *epd*_nul_): (0.4, 0.6) and (0.3, 0.5).

**Results:**

Compared with Fleming, the DESR was more likely to allow stage two of accrual and to reject the null hypothesis (*H*_nul_) after stage two, with rejection being more common with EPD parameters (0.4, 0.6) than (0.3, 0.5). Compared with Gehan, both DESR parameter sets accepted *H*_nul _in 15 trials after stage I compared with 8 trials by Gehan, with consistent conclusions in all 23 trials after stage II.

**Conclusions:**

The DESR may reject *H*_nul _when EPD rates alone are low, and thereby may improve phase II trial sensitivity to active, cytostatic drugs having limited response rates. Conversely, the DESR may invoke early stopping when response rates are low and EPD rates are high, thus shortening trials when drug activity is unlikely. EPD parameters should be chosen specific to each trial.

## Background

The increase in drugs available for study along with the human and resource costs for the conduct of clinical trials requires investigators to revisit trial design [1,2]. Nowhere is this more evident than in oncology, which must contend with more first-in-class drugs, longer development times, more drugs entering large phase III studies, and generally greater costs than other therapeutic areas [3]. In addition, the development of targeted drugs, which may induce limited tumour response, demands phase II trial designs which both minimize resource use and are sensitive and specific to signals of drug activity [4].

When response rate (RR) is used as a single primary endpoint, two sets of stopping rules have served as the basis for many prior two-stage phase II trials. The stopping rules of Gehan stop trials at the first stage when no response was observed [5]. The sample size for the first stage is based on a specified RR of interest and a beta error rate. If at least one response was observed, the second stage accrues using a sample size based on the desired standard error for the RR estimation and the number of responses observed in stage one. For the stopping rules of Fleming, the investigator specifies RR's of interest and disinterest as well as desired alpha and beta error rates [6]. Calculations determine the sample size in each stage and the minimum responses in stage one required to proceed to the second stage. The trial may be stopped after stage I of accrual to accept or reject the null hypothesis. Variations of the two-stage rules, such as those of Simon [7], have been designed to minimize the expected number of enrolled patients when drug is inactive. Despite the introduction of new study methods, the designs of Gehan, Fleming, and Simon still in common use [8,9].

Although RR remains the most common primary endpoint in phase II trials [8], disease stabilization may be a more appropriate endpoint for some agents and has also been associated with improved survival [10,11]. Similarly, a high rate of early progressive disease (EPD), defined here as progression at the first tumour measurement after initiation of treatment, correlates with poor survival [12,13]. Conversely, a low EPD rate may suggest drug activity, and could serve as a warning against early discard of a new agent. A combination of response and EPD as a multinomial endpoint would identify an active drug which produces a high response rate or low EPD rate.

Zee *et al *first derived stopping rules for a two-stage clinical trial with a multinomial endpoint of RR and EPD [14]. However, it was found that these stopping rules only achieved the desired power for an alternate hypothesis requiring sufficiently high RR *and *sufficiently low EPD, whereas the study had sought power for an alternate hypothesis allowing for either a favourable RR *or *a favourable EPD [15]. Recently, a new rule set [16], the Dual Endpoint Stopping Rule (DESR), was derived to address this problem. The new stopping rules offer the desired power as well as high rates of early stopping for drugs meeting the null hypothesis, but have not been applied to real data from phase II clinical trials. The objective of this paper is to compare the DESR with the stopping rules of Fleming and Gehan in a series of phase II trials as summarized by Dent *et al *[14,17]

## Methods

The Dual Endpoint Stopping Rule (DESR) for phase II trials with endpoints of response and early progressive disease (EPD) rates is described here briefly and in detail previously, where variations on the rules and sensitivity testing have been provided [16]. Specifically, DESR is based on testing of the following hypotheses:

where the response rates (*r*_nul_,*r*_alt_) and early progressive disease rates (*epd*_nul_,*epd*_alt_) of interest are prespecified. These hypotheses imply that a new drug would be considered of interest for further study if **either **the response rate, *r*, was sufficiently high **or **the early progressive disease rate, *epd*, was sufficiently low; it is not necessary that both outcomes occur.

After additional study parameters including the sample size for stage I (*n*_1_) and stage II (*n*_2_) of the trial and the desired alpha error rate and power are provided, stopping rules are generated by simulations performed using TreeAge Pro Healthcare software (Williamstown, Massachusetts) with the Borderline Value Method [16], which assumes that response and EPD rates of the desirable drugs are not better than *r *= *r*_alt _or *epd *= *epd*_alt_. With the DESR, the trial would be stopped at the first stage after *n*_1 _subjects are entered if *n*_*1r *_≤ *n*_1r-nul _and *n*_*1p *_≥ *n*_1p-nul_, where *n*_1r _and *n*_1p _are respectively the number of patients who responded and had early progression and *n*_1r-nul _and *n*_1p__-__nul _are thresholds of the DESR. Barring stopping, *n*_2 _more patients are recruited into the second stage. The null hypothesis will be rejected at the end of the second stage if *n*_*1r*_+ *n*_*2r *_≥ *n*_*1r-alt*_+ *n*_2r-alt _or *n*_1p_+ *n*_2p _≤ *n*_1p-alt _+ *n*_2p-alt_, where *n*_2r _and *n*_2p _are respectively the number of patients who responded and had early progression at stage II, *n*_1r-alt _+ *n*_2r-alt _represents the threshold number of responders required after stage II to conclude *H*_alt_, and *n*_1p-alt _+ *n*_2p-alt _is similarly the threshold for the stage I and stage II subjects with early progression to conclude *H*_alt_.

Data from two sets of phase II trials previously studied by Dent *et al *[17], were used to evaluate the DESR and compare it with stopping rules of Fleming and Gehan. The first set of these phase II trials was undertaken by the National Cancer Institute of Canada Clinical Trials Group, using the two stage stopping rule of Fleming. Trials were designed based on testing of hypotheses *H*_nul_: *r *≤ 5% and *H*_alt_: *r *≥ *20%*, which allows for continuation to the second stage of accrual (with *n*_2 _= 15) if one or more responses are observed among the first *n*_1 _= 15 patients. At the second stage, *H*_nul _is rejected if four or more responses are found. The second set of phase II trials was performed by the EORTC using the stopping rule of Gehan. The response rate of interest and beta error rate for the first stage were prespecified respectively as 20% and 0.05, which led to the sample size *n*_1 _= 14. Recruitment to the second stage occurs if at least one response is seen, with the size of *n*_2 _varying with the number of responses seen in the first stage in conjunction with a desired standard error rate. For comparison purposes, (*r*_nul_, *r*_alt_) was selected as (0.05, 0.2) to derive DESR thresholds. Based on the work of Zee *et al *and others [Zee, 1999;Sekine, 1999], two plausible parameter sets were selected for EPD, (*epd*_nul_,*epd*_alt_) = (0.6, 0.4) or (0.5, 0.3), to assess the impact of EPD on early stopping.

The alpha error rate and power used to derive DESR thresholds were respectively 0.05 and 0.8, although actual error rates vary from this according to the final thresholds selected by the program [Goffin, 2008]. The sample sizes for both stages were set the same as that in the Fleming rules or actual recruitment to the various EORTC studies when comparisons were made with the Fleming and Gehan stopping rules respectively.

## Results

Table 1 shows the thresholds of the DESR for the null and alternate hypothesis corresponding with the studies utilizing the rules of Fleming. The table is read along the first row of results as follows: With desired study parameters of *r*_nul _= 0.05, *r*_alt _= 0.2, *epd*_nul _= 0.6, *epd*_alt _= 0.4, alpha error 0.05, power 0.8, and two stages of accrual of 15 patients each, the trial would be stopped at the first stage to reject the drug (accept the null hypothesis) if there were 1 or fewer responding patients *and *8 or more patients with early progressive disease. Otherwise, the second stage would accrue, at the end of which the drug would be accepted (null hypothesis rejected) if 4 or more patients had responded to the drug or 14 or fewer progressed. This stopping rule would have an actual power of 0.796, alpha error of 0.025, and an expected number of 16.4 patients accrued if the drug under study was uninteresting (i.e. drug meeting *H*_nul_). Two pairs for the null and alternate hypothesis for *epd *are shown.

**Table 1:** Thresholds by DESR to compare with rules of Fleming (n_1 _= 15, n_2 _= 15, power = 0.8, alpha = 0.05)

Response		Early Progression		Stage 1 Drug Rejection		Stage 2 Drug Acceptance		Power	Alpha Error	**EN**_**nul**_**/PES**_**nul**_	**EN**_**alt**_**/PES**_**alt**_
***r***_**alt**_	***r***_**nul**_	***epd***_**alt**_	***epd***_**nul**_	***n***_**1r**_	***n***_**1p**_	***n***_**1r**_**+*n***_**2r**_	***n***_**1p**_**+*n***_**2p**_				
0.2	0.05	0.4	0.6	≤1/15	≥8/15	≥4/30	≤14/30	0.796	0.025	16.4/0.90	27.7/0.15
0.2	0.05	0.3	0.5	≤1/15	≥6/15	≥4/30	≤11/30	0.785	0.025	16.2/0.92	27.4/0.17

EN_nul _= expected number of patients accrued if a drug meets the criteria of the null hypothesis

EN_alt _= expected number of patients accrued if a drug meets the criteria of the alternate hypothesis

PES_nul _= probability of stopping the trial after stage I if a drug meets the criteria of the null hypothesis

PES_alt _= probability of stopping the trial after stage I if a drug meets the criteria of the alternate hypothesis

Thresholds for DESR trials sized to match the studies conducted under the rules of Gehan are shown in Tables 2 and 3. Table 2 gives values for *epd*_alt _= 0.4, *epd*_nul _= 0.6, while Table 3 gives values for *epd*_alt _= 0.3, *epd*_nul _= 0.5.

**Table 2:** Thresholds by DESR to compare with the rules of Gehan (*r*_alt _= 0.2, *r*_nul _= 0.05, *epd*_alt _= 0.4, *epd*_nul _= 0.6, power = 0.8, alpha = 0.05)

Study Size		Stage 1 Drug Rejection		Stage 2 Drug Acceptance		Power	Alpha Error	**EN**_**nul**_**/PES**_**nul**_	**EN**_**alt**_**/PES**_**alt**_
***n***_***1***_	***n***_**2**_	***n***_**1r**_	***n***_**1p**_	***n***_**1r**_**+*n***_**2r**_	***n***_**1p**_**+*n***_**2p**_				
14	1	≤1/14	≥8/14	≥2/17	≤7/17	0.835	0.089	14.1/0.89	14.9/0.14
14	3	≤1/14	≥8/14	≥2/17	≤9/17	0.855	0.101	14.3/0.89	17.0/0.008
14	4	≤1/14	≥8/14	≥3/18	≤9/18	0.797	0.051	14.4/0.89	17.4/0.14
14	5	≤1/14	≥8/14	≥3/19	≤9/19	0.787	0.041	14.6/0.89	18.3/0.14
14	6	≤1/14	≥8/14	≥3/20	≤9/20	0.776	0.034	14.7/0.89	19.2/0.14
14	9	≤1/14	≥8/14	≥3/23	≤11/23	0.814	0.04	15.0/0.89	21.7/0.14
14	10	≤1/14	≥8/14	≥3/24	≤12/24	0.829	0.046	15.1/0.89	23.9/0.008
14	11	≤1/14	≥8/14	≥4/25	≤12/25	0.779	0.035	15.2/0.89	24.9/0.008
14	13	≤1/14	≥8/14	≥4/27	≤14/27	0.817	0.032	15.4/0.89	25.2/0.14
14	15	≤1/14	≥8/14	≥4/29	≤14/29	0.807	0.032	15.7/0.89	26.9/0.14
14	16	≤1/14	≥8/14	≥4/30	≤14/30	0.8	0.027	15.8/0.89	27.8/0.14
14	18	≤1/14	≥8/14	≥4/32	≤16/32	0.829	0.026	16.0/0.89	29.5/0.14
14	20	≤1/14	≥8/14	≥5/34	≤17/34	0.809	0.0174	16.2/0.89	31.2/0.14
14	21	≤1/14	≥8/14	≥5/35	≤17/35	0.804	0.021	16.3/0.89	32.0/0.14
14	22	≤1/14	≥8/14	≥5/36	≤19/36	0.83	0.024	16.4/0.89	32.9/0.14
14	23	≤1/14	≥8/14	≥5/37	≤19/37	0.828	0.02	16.5/0.89	33.8/0.14

EN_nul _= expected number of patients accrued if a drug meets the criteria of the null hypothesis

EN_alt _= expected number of patients accrued if a drug meets the criteria of the alternate hypothesis

PES_nul _= probability of stopping the trial after stage I if a drug meets the criteria of the null hypothesis

PES_alt _= probability of stopping the trial after stage I if a drug meets the criteria of the alternate hypothesis

**Table 3:** Thresholds by DESR to compare with rules of Gehan (*r*_alt _= 0.2, *r*_nu__l _= 0.05, *epd*_alt _= 0.3, *epd*_nul _= 0.5, power = 0.8, alpha = 0.05)

Study Size		Stage 1 Drug Rejection		Stage 2 Drug Acceptance		Power	Alpha Error	**EN**_**nul**_**/PES**_**nul**_	**EN**_**alt**_**/PES**_**alt**_
***n***_***1***_	***n***_**2**_	***n***_**1r**_	***n***_**1p**_	***n***_**1r**_**+*n***_**2r**_	***n***_**1p**_**+*n***_**2p**_				
14	1	≤1/14	≥6/14	≥2/17	≤5/17	0.811	0.075	14.1/0.91	14.8/0.17
14	3	≤1/14	≥6/14	≥3/17	≤7/17	0. 773	0.043	14.3/0.91	16.5/0.17
14	4	≤1/14	≥6/14	≥3/18	≤7/18	0.775	0.038	14.3/0.91	17.3/0.17
14	5	≤1/14	≥6/14	≥3/19	≤7/19	0.773	0.034	14.4/0.92	18.2/0.17
14	6	≤1/14	≥6/14	≥3/20	≤7/20	0.769	0.031	14.5/0.91	19.0/0.17
14	9	≤1/14	≥6/14	≥3/23	≤9/23	0.805	0.038	14.8/0.91	21.5/0.17
14	10	≤1/14	≥6/14	≥4/24	≤9/24	0.753	0.026	14.9/0.91	22.3/0.17
14	11	≤1/14	≥6/14	≥3/25	≤9/25	0.797	0.033	14.9/0.91	23.2/0.17
14	13	≤1/14	≥6/14	≥4/27	≤11/27	0.791	0.023	15.1/0.91	24.8/0.17
14	15	≤1/14	≥6/14	≥4/29	≤11/29	0.789	0.024	15.3/0.91	26.5/0.17
14	16	≤1/14	≥6/14	≥4/30	≤11/30	0.787	0.028	15.4/0.91	27.3/0.17
14	18	≤1/14	≥6/14	≥4/32	≤13/32	0.811	0.023	15.5/0.91	29.0/0.17
14	20	≤1/14	≥6/14	≥5/34	≤13/34	0.781	0.015	15.7/0.91	30.7/0.17
14	21	≤1/14	≥6/14	≥5/35	≤13/35	0.779	0.013	15.8/0.91	31.5/0.17
14	22	≤1/14	≥6/14	≥5/36	≤15/36	0.803	0.016	15.9/0.91	32.3/0.17
14	23	≤1/14	≥6/14	≥5/37	≤15/37	0.804	0.014	16.0/0.91	33.2/0.17

EN_nul _= expected number of patients accrued if a drug meets the criteria of the null hypothesis

EN_alt _= expected number of patients accrued if a drug meets the criteria of the alternate hypothesis

PES_nul _= probability of stopping the trial after stage I if a drug meets the criteria of the null hypothesis

PES_alt _= probability of stopping the trial after stage I if a drug meets the criteria of the alternate hypothesis

### Comparison with the Stopping Rules of Fleming

The comparison of the DESR and Fleming stopping rules for first stage stopping and second stage rejection of the null hypothesis is shown in Table 4. The DESR was more permissive at the first stage. For the EPD parameters *epd*_alt _= 0.4, *epd*_nul _= 0.6, the DESR allowed 6 of the 10 studies stopped by the Fleming rule to continue to the second stage of accrual, all on the basis of an acceptably low EPD rate. Using the EPD parameters *epd*_alt _= 0.3, *epd*_nul _= 0.5, the DESR allowed only 2 of these same 10 studies to continue to the second stage. In all cases where the DESR allowed accrual to the second stage but the rules of Fleming did not, the final conclusions about activity of the drugs from DESR were unknown since there was no data from the second stage of the trials and we could find no published phase III trial and no U.S. Food and Drug Administration (FDA) indication for the drugs and diseases under study in these phase II trials.

**Table 4:** Comparison of the DESR and Fleming for Early Stopping and Rejection of *H*_nul_

	**Stage I (n = 15 unless indicated)**^**a**^		Stage II*		Stage I Stop?			Drug Activity		
**Trial**	**Res**	**Prog**	**Resp**	**Prog**	**Flem**	**DESR *epd *0.4/0.6**	**DESR *epd *0.3/0.5**	**Flem**	**DESR *epd *0.4/0.6**	**DESR *epd *0.3/0.5**

1	0	6			Y	N	Y	N	P	N
2	0	1			Y	N	N	N	P	P
3	0	10			Y	Y	Y	N	N	N
4	0	7			Y	N	Y	N	P	N
5	0	9			Y	Y	Y	N	N	N
6	0	14			Y	Y	Y	N	N	N
7	0/14	7/14			Y	N	Y	N	P	N
8	0	6			Y	N	Y	N	P	N
9	0	3			Y	N	N	N	P	P
10	0	12			Y	Y	Y	N	N	N
11	1	9			N	Y	Y	?	N	N
12	7	6	13/30	13/30	N	N	N	**Y**	**Y**	**Y**
13	1	6	1/25	12/25	N	N	Y	N	**Y**	N
14	1	6	3/30	13/30	N	N	Y	N	**Y**	N
15	2	4	2/30	8/30	N	N	N	N	**Y**	**Y**
16	1	6	4/29	12/29	N	N	Y	**Y**	**Y**	N

^a^Actual trial results.

DESR *epd *0.4/0.6 = Dual Endpoint Stopping Rule *epd*_alt _= 0.4, *epd*_nul _= 0.6

DESR *epd *0.3/0.5 = Dual Endpoint Stopping Rule *epd*_alt _= 0.3, *epd*_nul _= 0.5

Flem = result according to rules of Fleming

Prog = Progression

Res = Response

Y, N, P = Yes, No, Possible

While six studies (Trials 11 through 16) were permitted to accrue to the second stage according to the Fleming rule, one study (Trial 11) was stopped by the investigators and this same study would have been stopped at stage one by the DESR. In the remaining five studies, *H*_nul _was rejected at end of study by the Fleming rule in two (12 and 16). By comparison, for the EPD parameters *epd*_alt _= 0.4, *epd*_nul _= 0.6, the DESR rejected *H*_nul _in all five trials at the end of stage II as a result of acceptable rates of EPD. Conversely, for the EPD parameters *epd*_alt _= 0.3, *epd*_nul _= 0.5, the DESR stopped three of the five trials at stage I, and rejected *H*_nul _after stage II in two trials (studies 12 and 15), with one consistent with the conclusion from Fleming rule (Trial 12). The differences again lay in the threshold for *epd *in the hypotheses under testing, with the EPD parameter set (*epd*_alt _= 0.3, *epd*_nul _= 0.5) requiring a lower observed rate of EPD for rejection of *H*_nul _than the EPD parameter set (*epd*_alt _= 0.4, *epd*_nul _= 0.6). In all cases where the DESR rejected *H*_nul _but Fleming did not, we found no phase III trial to confirm or deny drug activity, and no disease-specific FDA indication was found. The same lack of confirmation was found for study 16 which rejected *H*_nul _by the Fleming rule but not by the DESR with EPD parameters *epd*_alt _= 0.3, *epd*_nul _= 0.5.

### Comparison with the Stopping Rules of Gehan

Comparing the DESR rules based on two sets of EPD parameters in the cohort of phase II trials conducted under the Gehan design, the choice of null and alternate values for *epd *did not alter the likelihood of early stopping or rejection of the null hypothesis by the DESR, in part as a result of consistently high rates of EPD in trials 1-15 (see Table 5).

**Table 5:** Comparison of the DESR and Gehan for Early Stopping and Rejection of *H*_nul_

	**Stage I (n = 14)**^**a**^		**Stage II**^**a**^		Stage I Stop?			Drug Activity		
**Trial**	**Res**	**Prog**	**Resp**	**Prog**	**Gehan**	**DESR *epd *0.4/0.6**	**DESR *epd *0.3/0.5**	**Gehan**	**DESR *epd *0.4/0.6**	**DESR *epd *0.3/0.5**

1	0	14	0/23	20/23	Y	Y	Y	N	N	N
2	0	10	0/24	18/24	Y	Y	Y	N	N	N
3	0	9	0/17	10/17	Y	Y	Y	N	N	N
4	0	8	0/36	23/36	Y	Y	Y	N	N	N
5	0	11	0/19	14/19	Y	Y	Y	N	N	N
6	0	13	0/23	20/23	Y	Y	Y	N	N	N
7	0	11	0/15	12/15	Y	Y	Y	N	N	N
8	0	11			Y	Y	Y	N	N	N
9	1	12	2/34	23/34	N	Y	Y	N	N	N
10	1	8	2/27	16/27	N	Y	Y	N	N	N
11	1	10	1/19	12/19	N	Y	Y	N	N	N
12	1	10	2/25	17/25	N	Y	Y	N	N	N
13	1	10	1/20	15/20	N	Y	Y	N	N	N
14	1	9	1/17	11/17	N	Y	Y	N	N	N
15	1	12	1/18	16/18	N	Y	Y	N	N	N
16	4	9	7/29	14/29	N	N	N	Y	Y	Y
17	8	2	20/34	3/34	N	N	N	Y	Y	Y
18	5	4	7/32	13/32	N	N	N	Y	Y	Y
19	4	4	12/37	14/37	N	N	N	Y	Y	Y
20	4	3	7/30	11/30	N	N	N	Y	Y	Y
21	5	4	9/36	9/36	N	N	N	Y	Y	Y
22	3	4	6/27	10/27	N	N	N	Y	Y	Y
23	11	1	27/35	3/35	N	N	N	Y	Y	Y

^a^Actual trial results.

DESR *epd *0.4/0.6 = Dual Endpoint Stopping Rule *epd*_alt _= 0.4, *epd*_nul _= 0.6

DESR *epd *0.3/0.5 = Dual Endpoint Stopping Rule *epd*_alt _= 0.3, *epd*_nul _= 0.5

Prog = Progression

Res = Response

Y, N, P = Yes, No, Possible

Of the 23 trials conducted using the Gehan stopping rules, eight would have been stopped at stage I for acceptance of H_nul _by both Gehan and the DESR. In actuality, investigators continued seven of those trials (studies 1-7) through the second stage, although in all cases the studies were ultimately negative.

In the other 15 trials (9 to 23), accrual to the second stage was permitted under the stopping rules of Gehan. Of these, seven trials would have been stopped at the first stage by the DESR as a result of high *epd *rates in conjunction with only a single responding subject in each trial, and in all seven of these trials the rules of Gehan found the same results after accrual of the second stage (i.e., *H*_nul _accepted). In the final eight trials, *H*_nul _was rejected after the second stage by both the Gehan stopping rule and the DESR.

## Discussion

The DESR uses the signal provided by the rate of early progressive disease in an attempt to better discern drug effectivess compared with response alone [16]. It has been demonstrated that rules can be generated that meet the specified alpha error rate and power; this study assesses the relevance of the DESR when applied to actual patient data from phase II clinical trials [17].

Compared with the stopping rules of Fleming, the DESR was more likely to allow accrual of the second stage. This was more common with the rules specifying *epd*_nul _= 0.6 than *epd*_nul _= 0.5, as a higher EPD rate was tolerated without early drug rejection in the former case. At the second stage, the DESR with design parameters *epd*_alt _= 0.4, *epd*_nul _= 0.6 rejected *H*_nul _more frequently than either the Fleming stopping rules or the DESR with parameters *epd*_alt _= 0.3, *epd*_nul _= 0.5.

A somewhat different result was seen when comparing the DESR and the stopping rules of Gehan. In this instance, 15 studies were stopped at the first stage by the DESR (using both *epd *design parameter pairs), while only 8 were stopped by Gehan at the first stage, with high rates of EPD triggering the more frequent early stopping by the DESR. The discrepant seven studies ultimately accepted *H*_nul _at the end of the second stage under Gehan stopping rules. For the remaining eight studies allowed to continue to the second stage by the Gehan stopping rules and the DESR, conclusions on *H*_nul _were consistent between the rules.

The DESR is designed to find drugs that have either a desirable rate of response or a desirably low level of early progression. However, because it is designed to find the 'good' drugs among a mixed (50/50) population of drugs having either good response or early progression rates, it appears to require a higher response rate at the end of stage one to allow recruitment of stage two than that required if response is considered in isolation. For this reason, compared with the Gehan stopping rule, the DESR was more likely to stop trials after the first stage of accrual despite a single response being observed in stage I. Conversely, as noted above, the DESR was less likely than the Fleming rules to stop a study at stage I despite a lack of any response, as EPD rates were low enough that the drugs under study might have met the specified level for an interesting agent.

For trials in which response is the clear priority, a set of rules devoted to response only may be more appropriate. However, in the present age of molecularly targeted anti-cancer agents, the likelihood of an investigational agent inducing tumour shrinkage or preventing tumour growth is often unclear prior to initiating phase II studies.

In the absence of suitable rules, examples are readily found of investigators setting a primary endpoint of response, a drug failing to meet that response, but the drug being declared interesting for further study based on other desirable characteristics [18,19]

Other authors have investigated the use of multiple endpoints in phase II trials. Zee *et al *generated a set of stopping rules similar to the DESR, but later found that the rules generated had poorer power than intended [14,15]. However, results for the comparisons between DESR and the stopping rules of Zee with Gehan's stopping rules were very similar in the same data set [17]. Although only the design parameter pair *epd*_alt _= 0.4, *epd*_nul _= 0.6 was considered in the paper which applied their rules [17], both the DESR and the stopping rules of Zee *et al *stop the first 15 trials at stage I and reject *H*_nul _after stage II in the remaining trials, with high EPD rates being the common reason for early stopping. Conversely, considering drugs studied under the Fleming stopping rules, the DESR was less likely to accept *H*_nul _at the end of stage I, and so to recruit to stage II. The conclusions at the end of stage II were more difficult to compare, as many of the actual trials did not recruit to the second stage. While the DESR remained more likely to reject *H*_nul _for the design parameter pair *epd*_alt _= 0.4, *epd*_nul _= 0.6, it may have been less likely to reject *H*_nul _with the pair *epd*_alt _= 0.3, *epd*_nul _= 0.5, suggesting the sensitivity of the results to changes in the design EPD parameters.

In an analogous paper, Panageas *et al *consider a rule set where response is divided into complete and partial response, and levels of interest and disinterest are again specified for the null and alternate hypothesis [20]. This rule set is potentially attractive for highly responsive cancers such as germ cell tumours, where complete responses are more frequent. However, it may be less applicable in the setting of most phase II trials involving previously treated malignancies and targeted drugs with uncertain tumour effects. In this setting, complete responses may be infrequent, and modest response rates or non-progression may suggest drug activity and lead to drug approval [8]. A slight modification to this design can be made which substitutes response and stable disease for complete response and partial response, similar to the DESR design. However, the study power calculated when using the Panageas design may actually be overestimated, thus underestimating the number of patients needed. This is because power is calculated assuming *r*_alt _and *epd*_alt _are simultaneously at the exact minimum response rate and maximum early progressive disease rate of interest for further study for the novel agent. The DESR design using the borderline method varies *r*_alt _and *epd*_alt _while maintaining power. Both endpoints do not have to be simultaneously at the boundary of interest, potentially giving a more accurate estimate of statistical power.

One limitation to the present study is that it applies arbitrary *epd*_alt _and *epd*_nul _pairs to existing data. Individualized *epd *rates may be more relevant to a given drug and give different results, although the pairs chosen were felt to be commonly plausible. Additionally, although the results presented are only for trials in which the *H*_nul _for response rate is 0.05, the DESR method can be implemented for trials with higher null response rates. This comparison was not performed due to a critical lack of published phase II trials which present response and EPD rates at both stage I and II. It is also unknown whether actual efficacy might have been seen when the DESR rejected *H*_nul _but the Fleming rule did not, as subsequent phase III studies were not conducted.

## Conclusion

In conclusion, while the number of trials in our study is small, different patterns of early stopping and final rejection of *H*_nul _are evident with the addition of EPD as an endpoint. With limited follow-up in terms of phase III studies, the final benefit in terms of drug development is not certain. However, the DESR may shorten studies where response rates are low but high EPD rates suggest the ultimate efficacy will be poor. Conversely, the DESR will allow accrual to the second stage in the absence of response when there are few patients with EPD, and this may allow more sensitive detection of drug activity. Based on the comparisons in this paper, the *epd*_alt _= 0.3, *epd*_nul _= 0.5 pair appears to offer the better balance of these outcomes, but the design parameters for a particular trial should be individualized.

## Competing interests

The authors declare that they have no competing interests.

## Authors' contributions

JRG designed the study, programmed simulations, analyzed data, and drafted the manuscript. GRP designed the study, analyzed data, and drafted the manuscript. DT designed the study, analyzed data, and drafted the manuscript. All authors read and approved the final manuscript.

## Pre-publication history

The pre-publication history for this paper can be accessed here:

http://www.biomedcentral.com/1471-2288/11/95/prepub
